# Arthroscopic guided biopsy and radiofrequency thermoablation of a benign neoplasm of the tibial spines area: a treatment option

**DOI:** 10.1186/1471-2474-13-52

**Published:** 2012-04-04

**Authors:** Carmine Zoccali, Giuseppe Teori, Nicola Salducca, Berardino Di Paola, Ezio Adriani

**Affiliations:** 1 Oncological Orthopedics Department, Muscular-skeletal Tissue Bank, IFO - Regina Elena National Cancer Institute, Via Elio Chianesi 53, Rome, Italy; 2 Oncological Orthopedics Department, IFO - Regina Elena National Cancer Institute, Via Elio Chianesi 53, Rome, Italy; 3 "Mater Dei" Medical Sport Center, Rome, Via Elio Chianesi 53, Rome, Italy

**Keywords:** Radiofrequency thermoablation, Tibial spines, Osteoid osteoma, Chondroblastoma, Arthroscopic guided biopsy, I declare that we have no commercial interest in the subject under study and that we have not received any financial or material support

## Abstract

**Background:**

Lesions located in the area of the tibial spines are rare. In most cases, treatment follows histological diagnosis, but when imaging and clinical data are considered to be "very" characteristic for benign lesions, such as chondroblastoma or osteoid osteoma, treatment may be performed without biopsy. Traditional curettage requires opening the joint, which presents a high risk of contamination of the joint itself and surrounding structures, such as the popliteal area, with possible contamination of the neurovascular bundle when performing curettage with the posterior approach. In this case, the re-excision of a local recurrence would be extremely difficult.

**Results:**

We describe a technique using arthroscopic guidance for radiofrequency thermoablation of a benign lesion in the tibial spines area. We report on an illustrative case. The patient so treated, reported immediate relief from the pain, and after two weeks, was free of pain. The biopsy performed before the treatment confirmed the radiological diagnosis of chondroblastoma. At one year of follow-up, the patient is without pain, with a 0-130°range of motion, has no activity limitations and is apparently free of disease.

**Conclusion:**

This technique allows a radiofrequency thermoablation of a lesion in the tibial spines area and in the posterior tibial surface to be performed without opening the joint, monitoring the tibial plateau surface, probably decreasing the risk of cartilage damage. Unfortunately, in the case presented, the high pressure from the arthroscopy's pump broke the tibial plateau surface creating a communication to the tibial tunnel used for thermoablation.

## Background

Tumors located in the tibial spines area are unusual. Biopsy is difficult because of the high risk of contamination of the joint, therefore, it should be done under guidance of a CT-scan. In some cases where radiologic imaging is characteristic for benign lesions, such as chondroblastoma or osteoid osteoma, treatment may be performed at the same time as biopsy, ideally with an expert pathologist available for intraoperative histology.

This paper reports an unusual case of chondroblastoma located in the tibial spines and describes an arthroscopic technique used to guide an extra-articular biopsy, followed immediately by Radiofrequency Thermoablation (RT) of the tumor.

## Results and discussion

### Case report and technique description

A 28 year-old male was referred to our department complaining of pain in his right knee for the last four years. During that time, he had undergone three arthroscopies for suspected meniscopathy without any results.

**Figure 1:**
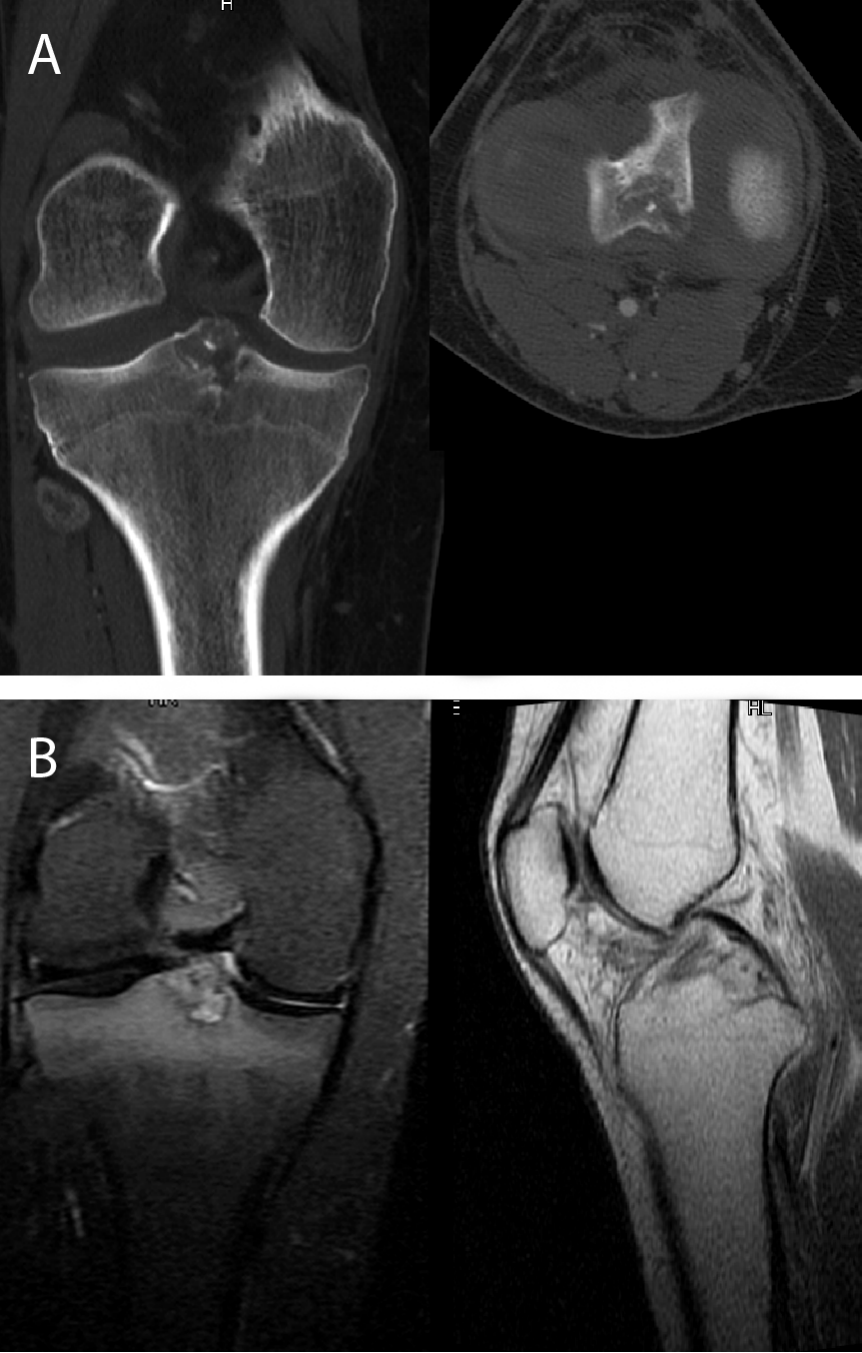
**A-B CT and MRI scan showing an osteolytic lesion in the spines area**.

A new CT-scan and a MRI-scan of the patient's right knee evidenced the presence of a neoformation located in the proximal tibia, posterior to the insertion of the cruciate ligaments, which had been misinterpreted at the previous scans when it was not clearly visible (Figure 1). The imaging strongly suggested a benign chondroblastoma. Due to the position of the disease a curettage by posterior approach was considered but this would have caused possible contamination of the popliteal neurovascular bundles in case of local relapse, thus an arthroscopic-guided RT with intraoperative biopsy of the lesionwas recommended.

The exploratory arthroscopy confirmed the absence of intra-articular disease, therefore a biopsy was carried out and an arthroscopic-guided RT was performed. A frozen section was not obtained because the imaging and clinical aspect were considered clear.

**Figure 2:**
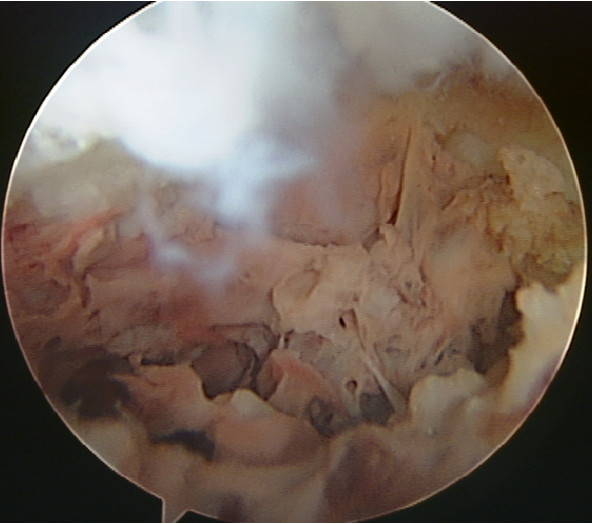
**Arthroscopic view showing the aspect of the spines area after radiofrequency thermoablation and tibial surface rupture**.

**Figure 3:**
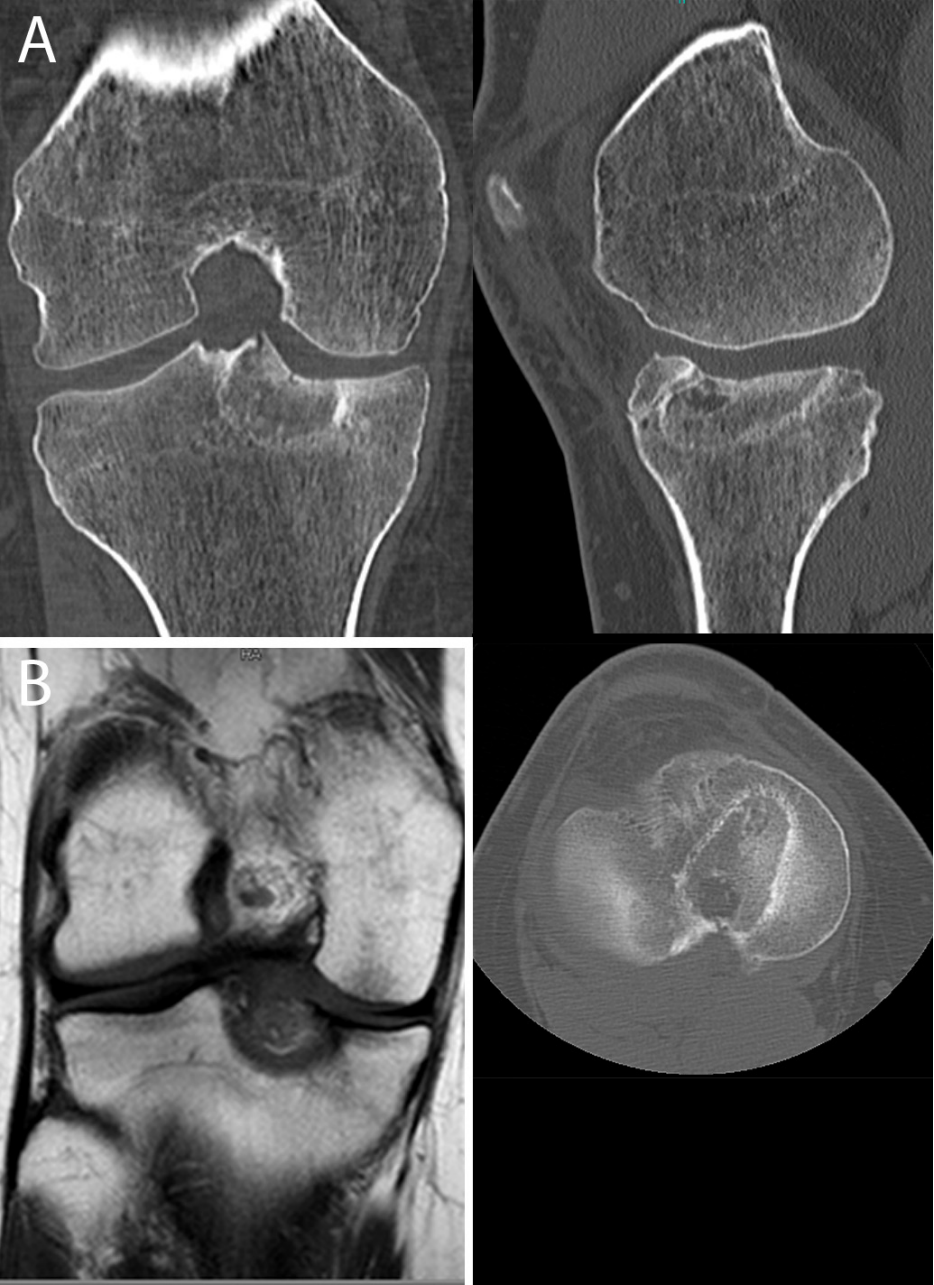
**A, B: CT and MRI scan performed at one year of follow-up**.

Unfortunately, after the procedure, the high pressure from the arthroscopy's water pump broke the tibial plateau surface and created a communication to the tunnel used for the procedure (Figure 2). Nevertheless, at one year of follow-up, the patient is without pain, with a 0-130° range of motion, has no activity limitations and is apparently free of disease, according to CT and MRI scans (Figure 3).

The patient gave his permission for publication of his images and his case

### Technical note

**Figure 4:**
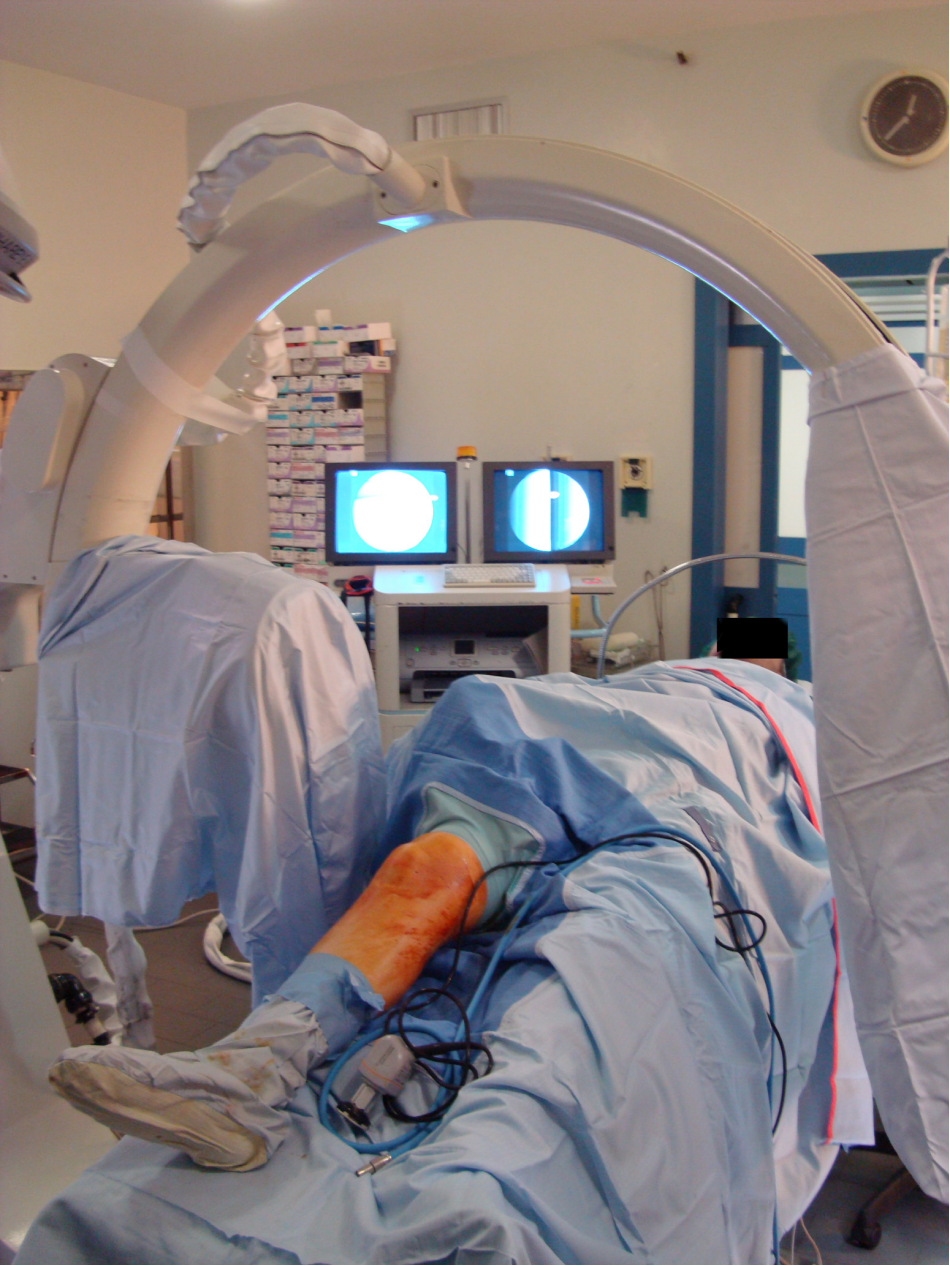
**Surgical placement with the C-arm placed in the lateral view**.

For knee arthroscopy, the patient is positioned as required by the surgeon. In this case, the patient was supine with hip and knee flexed to facilitate the access to the knee joint. An X-ray image intensifier was located as shown in Figure 4 to allow a lateral view of the knee during the arthroscopy.

Access was provided via the routine parapatellar inferior anterolateral and anteromedial portals, avoiding the fat pad. Exploratory arthroscopy identified the tibial spines.

**Figure 5:**
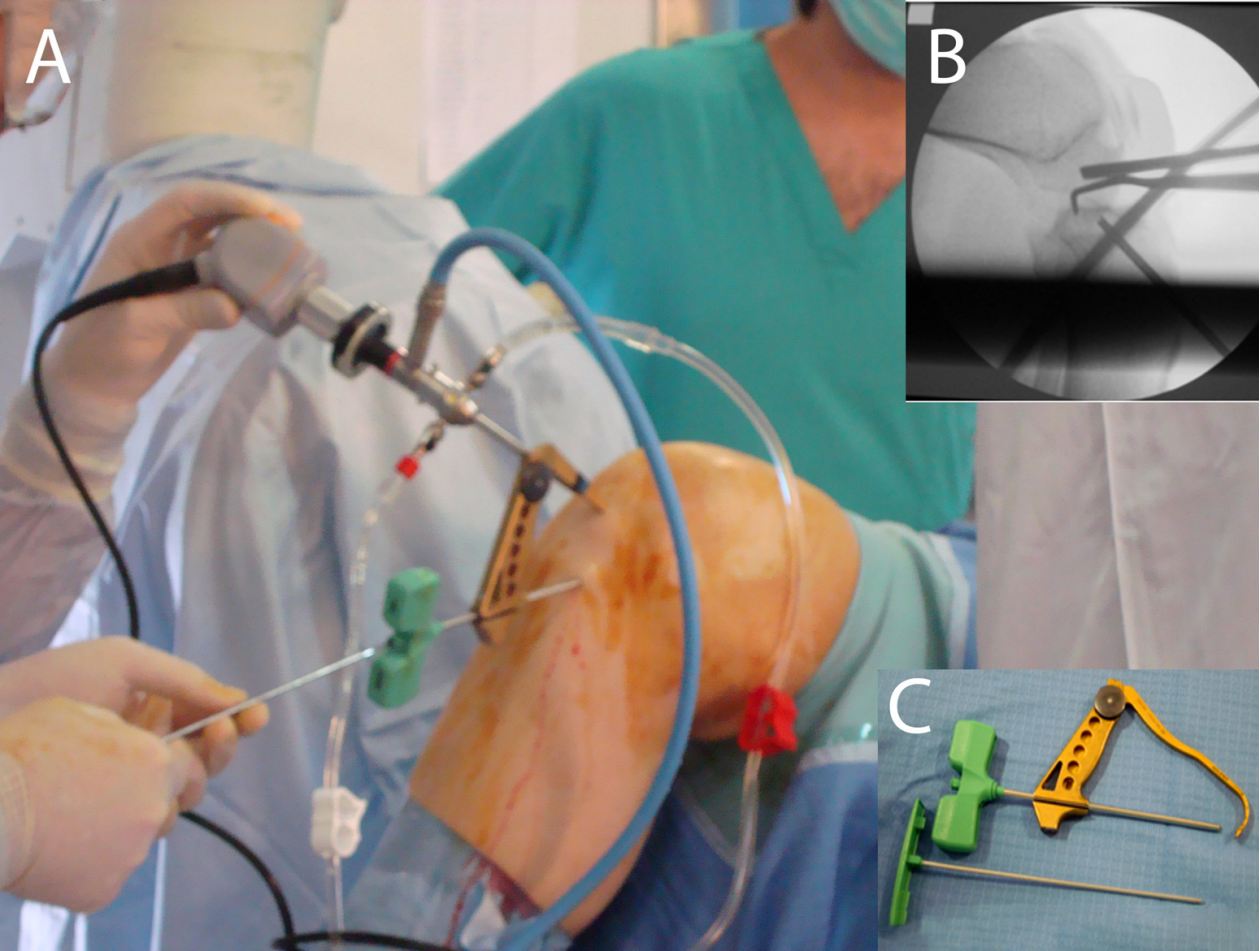
**A: The biopsy is performed through the access usually used to reconstruct the anterior cruciate ligament**. B: X-rays intensifier Check showing the correct placement of the biopsy trocar. C: the biopsy trocar and the guide used.

A drill guide was then put through the medial port (Figure 5A, C), aimed at the tibial spines, then the biopsy trocar was inserted to the margin of the neoformation. Placement was confirmed by imaging with the X-ray intensifier, and the biopsy performed (Figure 5A, B).

Following extraction of the biopsy specimen, the same access was used for the RF thermoablation of the tumor. The system used was a monopolar "angiodynamics RITA Model 1500X^®^" with a "StarBurst^®^"multi-tined expandable probe with seven tips.

The radiofrequency probe was placed at the lesion; placement and expansion were again confirmed radiographically by the C-arm, and the RT was done, reaching a temperature of 100°C for ten minutes.

The arthroscopy permitted the monitoring of the articular surface during the entire procedure and no changes were visible. Unfortunately, in this case, the high pressure from the arthroscopy's water pump broke the tibial plateau surface after the RT, creating a communication between the joint and the access tunnel used for the treatment (Figure 2).

Fortunately, this did not influence the results, and after one year of follow-up no sign of any recurrence is present at MRI and CT-scans (Figure 3). It is also possible to note the wide effect of the treatment; RT was not limited to the lesion area but also took in the surrounding tissue, thus in the postoperative imaging the treated area looks bigger than the original disease area. This explains the possible advantage of a direct view of the cartilage, allowing the procedure to be stopped in case of any visible change in the aspect of the cartilage, and to pull the probe slightly backward and successively start the radiofrequency thermoablation again.

The preferred approach to biopsy of lesions in the tibial spines area is by CT-guidance, using an access point, which avoids joint space to decrease the risk of neoplastic contamination. Nevertheless, in selected cases, radiologic findings are so strongly characteristic of benign lesions that biopsy and ablative therapy may be planned as a single, two-step operative procedure. Here we have presented a case in which the imaging and clinical data (very slow growth) was highly suggestive of chondroblastoma, a benign tumor typical of the epiphysis. In this case, treatment was carried out at the same time as the biopsy.

Benign chondroblastomais often localized in epiphyseal areas. It is usually found in patients between 10 and 20 years of age, and less frequently in patients between 21 and 30 years of age. The imaging is typical of an osteolytic rounded lesion, with smooth, well-defined borders and sometimes calcifications. On CT and MRI scans, the lesion appears to be parenchymatous, occasionally with cystic cavities and fluid levels [1].

The gold standard in the treatment of chondroblastoma is curettage, with or without the use of local adjuvants.

When the lesion is located under the anterior part of the tibial articular surface the surgical approach is anterior; when the lesion is located under the tibial spines area or posterior, the approach should be posterior because of the presence of cruciate ligaments, identifying the popliteal neurovascular bundles.

Today, RT has become a minimally invasive alternative to surgery [2], eliminating the need for capsule opening and the accompanying increased risk of joint and popliteal area contamination and recurrence, which has historically been very difficult to treat. The approach proposed here allows treatment of the lesion without opening the joint so that any possible recurrence in the tibia would be less difficult to treat, either by curettage or by repeat radiofrequency thermoablation.

A CT-guided RT is probably easier than the arthroscope-guided procedure described herein, because it allows for more accurate verification of the correct placement of the RT probe. However, CT-guidance does not allow for the cartilage surface to be directly monitored during the RT, otherwise possible with the arthroscopic procedure described here, probably decreasing the risk of damage to these tissues during the ablation therapy. Unfortunately, no studies about the changes induced by RT are available in literature, nevertheless, in our case, no changes were noted during the procedure. If any variation in the aspect and color of the cartilage were noted, radiofrequency thermoablation could be stopped and restarted after pulling the probe backward and positioning it farther from the cartilage or after decreasing the working temperature.

Unfortunately, in the case presented here, the high pressure from the arthroscopy's water pump broke the tibial joint surface and created a communication between the joint and the disease area. This happened because the water pressure was not decreased during and after the procedure, rather than as a direct consequence of the treatment. The rupture occurred after RT when theoretically the tumoral cells had died, therefore local relapse risk is, definitely, less than that of traditional curettage: the high temperature achieved by RT created a significant necrosis, so the patient remains disease-free at one year of follow-up.

Attention must be paid to minimize the intra-articular water pressure during and after the procedure of arthroscope-guided radiofrequency thermoablation.

The arthroscopic procedure can be performed in any hospital where arthroscopic and RF instruments are available. However, the CT-guided procedure requires, in addition to RF instrumentation, an anesthesia-supported CT suite, which is not widely available. Indeed both procedures require general or epidural anesthesia.

## Conclusions

The main advantage of our technique is to allow the treatment of benign lesions in the tibial spines area and in the posterior part of the tibial articular surface where a posterior approach is needed without opening the joint for curettage. Moreover, this technique should permit the cartilage to be directly monitored during the procedure, and because of its safety and relative ease, it can be performed by surgeons at varying levels of expertise in arthroscopic procedures. Nevertheless, more studies need to be conducted to describe the possible cartilage damage.

The upper size limit of the lesions potentially treated by RF depends on the opening of the probe tips technically possible up to 5 cm; nevertheless this size cannot be obtain within the bone and the radiofrequency application would not be uniform, therefore, in our opinion, lesions bigger than 3 cm in diameter should not be treated.

A fundamental requirement of this treatment is that it must only be done after histologic confirmation of a benign lesion, or when radiologic imaging is strongly characteristic of a benign lesion.

## Consent

The patient has given his consent for the publication of his case and his images.

## Competing interests

The authors declare that they have no competing interests.

## Authors' contributions

CZ drafted the manuscript, GT designed the procedure, NS performed the procedure, BDP revised the paper, EA performed the procedure and supervised the manuscript. All authors read and approved the final manuscript.

## Pre-publication history

The pre-publication history for this paper can be accessed here:

http://www.biomedcentral.com/1471-2474/13/52/prepub

## References

[B1] Springfield DS Capanna R Gherlinzoni F Picci P Campanacci M Chondroblastoma A review of seventy cases J Bone Joint Surg Am 1985 67 5 748 755 3997927

[B2] Rybak LD Rosenthal DI Wittig JC Chondroblastoma: radiofrequency ablation--alternative to surgical resection in selected cases Radiology 2009 251 2 599 604 Epub 2009 Mar 20 10.1148/radiol.2512080500 19304917

